# Beyond the Unknown: A Broad Framing for Preparedness for Emerging Infectious Threats

**DOI:** 10.4269/ajtmh.22-0341

**Published:** 2022-10-03

**Authors:** Rebecca C. Christofferson, Stephania A. Cormier

**Affiliations:** ^1^ School of Veterinary Medicine, Louisiana State University, Baton Rouge, Louisiana;; ^2^ College of Science, Louisiana State University and Pennington Biomedical Research Center, Baton Rouge, Louisiana

## Abstract

There have been multiple instances of novel pathogen emergence that have affected the health and security of the global community. To highlight that these novel pathogens presented a clear danger to public health, the WHO included “Disease X” on their list of priority pathogens in 2018. Indeed, since the emergence of SARS-CoV-2, Disease X has been pointed to as the looming threat of “the next big thing.” However, developing surveillance and preparedness plans with Disease X as the linchpin is too narrow and ignores a large swath of potential threats from already identified, often neglected diseases. We propose instead the idea of “Disease f(x)” as a preferred call to arms with which to prioritize research and programmatic development. The common mathematical notation f(x) represents the knowledge that outbreaks are a function of many variables that define the transmission trajectory of that pathogen. Disease f(x) exploits commonalities across pathogen groupings while recognizing that emergences and outbreaks are fluid and that responses need to be agile and progressively tailored to specific pathogens with cultural and regional context. Adoption of this mindset across sectors, including biotechnology, disaster management, and epidemiology, will allow us to develop more efficient and effective responses to address the next major infectious threat.

## NOT JUST DISEASE X

In the 21st century, there have been several major outbreaks of novel pathogens, such as SARS-CoV-1, Middle Eastern respiratory virus, severe fever with thrombocytopenia syndrome virus, and SARS-CoV-2.^1^^–^^4^ Novel pathogens continue to pose a significant threat, especially as the global community continues to be more interconnected and as human activities drive us and animal populations to closer interaction, increasing the risk for spillover events.^5^^,^^6^ In 2018, the WHO codified this concept of an unknown threat when it included “Disease X” on its list of priority pathogens.

The WHO formally defined Disease X as representing “the knowledge that a serious international epidemic could be caused by a pathogen currently unknown to cause human disease.”^7^ This unprecedented move acknowledged that the pathogenome of the world still held many unknowns, and encouraged initiatives aimed at enhanced surveillance and preparedness. By including Disease X on the priority pathogen list, the WHO signaled that research and development should account for the unknown threat. As SARS-CoV-2 and previously emergent novel pathogens have shown us, the novel pathogen poses more than just a theoretical threat. The pandemic has brought increased references to Disease X in preparing the global community for subsequent threats. But calls to action for pandemic preparedness under the banner of Disease X are too narrowly focused, and a balance must be struck between accounting for unknown dangers and maintaining focus on already identified, often neglected infectious diseases of public health importance.

In fact, some of the most significant outbreaks in the past 20 years have been from previously identified pathogens, such as cholera in Zimbabwe 2008; pandemic swine flu in 2009; chikungunya and Zika viruses in South and Central America 2014 and 2015, respectively; Ebola virus in West Africa 2014–2015; and yellow fever virus in Brazil 2016.^8^^–^^13^ In addition, during the writing of this piece, there has been identification of monkeypox virus in the Europe and elsewhere and detection of polio virus in Mozambique after 30 years, detection in the UK after 40 years as well as the United States (New York).^14^^–^^17^ Introductions, expansions, and reemergence events highlight that discreet characterization of known pathogens is also not sufficient. Some pathogens seem to be adequately characterized, but what we know may in fact become insufficient given significant changes to the ecology of the pathogen. For example, changes due to urbanization and deforestation encourage spillovers and introductions into new geographies; climate change leads to expansion of vectors and their associated pathogens; and social issues (e.g., the rise in antivaccination movements or forced migration) alter the demographics of human communities.^18^ All these factors essentially provide more opportunities for pathogen contact with susceptible hosts, affording the means for emergence and the potential for fulminant outbreaks. Thus, although there is an important need for virus hunters to continue to discover the identities of potential Disease X pathogens, prospective and steady basic research and public health preparedness needs a broader framing for efficient and expedient allocation of resources and ultimately translation of results into programmatic development and optimization.

### A broader, One Health-aligned framework.

One Health is the understanding that health of humans, animals, and the environment are interconnected and is a critical paradigm for framing future research for optimized planetary health.^19^ One Health is the context in which specific problems should be set to formulate holistic and balanced solutions. One Health encompasses myriad things currently threatening global health, including (but not limited to) infectious diseases, noncommunicable diseases, environmental disasters, and climate change. Disease X as a focus for surveillance does not intuitively put things into the One Health paradigm because it focuses on the identity of a pathogen and, in some respects, whether it has been previously identified or not.

Developing subframeworks that are One Health aligned but that address specific aspects of health focuses efforts, identifies the most relevant stakeholders, and leads to balanced and efficient responses. As an example, we discuss one such subframework to address emerging infectious threats specifically and associated preparedness and response. We propose the framework of Disease f(x), where f(x)—a commonly used mathematical notation—is meant to symbolize that public health crises caused by infectious diseases are complex and multivariate.

We propose defining Disease f(x) as “the knowledge that epidemic or pandemic potential of a pathogen—known or unknown—cannot be defined without understanding the multitude of variables that affect the biological processes of a pathogen, including transmission, ecology, pathogenesis, zoonotic potential, and the interaction with host(s) and the environment.” Disease f(x) would promote basic and operational support research by clearly expressing at the outset that the factors that define potential risk due to a pathogen—that is, the complete set of variables that determine the transmission dynamics of a pathogen’s spread—should be addressed in tandem. We posit that such an approach would provide an improved basis on which surveillance and response systems can be built. As it encompasses the intrinsic and extrinsic factors that affect the biology of the pathogen, it is necessarily One Health aligned. Disease f(x) can be used to frame research and development, a priori preparedness, and response schemes.

In addition to specific therapeutics development, pandemic research and development should include and prioritize the transmission ecology of pathogens, both natural and anthropogenic. As SARS-CoV-2 has demonstrated, social and political pressures, as well as global equity issues have all contributed to the persistence of the virus, even when therapeutics were deployed in record time.^20^^,^^21^ Deficiencies in understanding of the transmission mode(s) early in the pandemic delayed masking and other nonpharmaceutical intervention recommendations which were further exacerbated by delayed and/or poor science communication.^22^^,^^23^ Further, there are a multitude of viruses for which diagnostic capabilities are currently non-existent or severely lagging, as was highlighted by the West African Ebola virus (2014–2015) outbreak and the Zika virus outbreak in South America (2015–2016).^24^ Additionally, a lack of quickly available, sensitive, and specific diagnostics may create a reliance on clinical syndrome for diagnosis, which creates an additional issue of possible misdiagnosis.^25^

Critically, by shifting the novelty of the “next big thing” from Disease X, potential threats from already identified (possibly neglected) pathogens will not be discounted. Additionally, Disease f(x) is a platform with which to lobby funders and review panels to support research not only to surveil for Disease X—because virus hunting remains important—but also to characterize pathogens comprehensively, especially those with zoonotic potential that seem innocuous but may just be uncharacterized and underdiagnosed. Relatedly, Disease f(x) lays the groundwork for supporting steady investigation into the ways that transmission may be altered due to external forces such as climate change or human behavior, even though pathogen identity may remain unchanged. For example, understanding how climate change may affect transmission could inform vector control resource allocation and surveillance efforts.^26^ In addition, the rise of the antivaccine movement has called into the question the control of preventable disease.^27^ Any security we may feel for current responses to infectious threats may become invalid given enough perturbation of transmission parameters. Thus, preparedness based on the holistic system of Disease f(x) is not only necessary for framing initial preparedness but also for staying prepared amid global changes.

**Figure 1.**
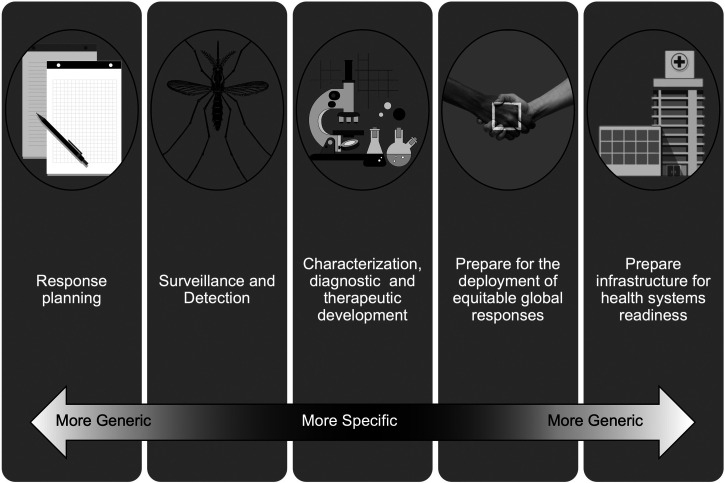
Championing a broad framework for preparedness that is One Health aligned allows for development of flexible programs that are progressively specific, as necessary, but exploits common aspects of pathogen transmission to allow for generic planning programs and response protocols.

Finally, Disease f(x) promotes thinking about responses with some agnosticism, by identifying those factors that are common across pathogens or pathogen groups and that do not specifically rely on identity to drive response elements. Specificity of pathogen identity remains obviously critical for efficacious therapeutics and diagnostics, but by cataloguing and exploiting commonalities, we create an advantage as threats emerge. Critical but relatively generic response elements, which are largely dependent on transmission modalities rather than individual pathogen identity, should be quickly deployed at the beginning of any potential outbreak. For example, clinical and public health workforce preparedness may depend on whether they are prepared for a bloodborne pathogen versus a respiratory virus. When dealing with vector-borne diseases, communications regarding vector avoidance behavior can be tailored to the relevant, shared arthropods. On the other hand, by specifically understanding how the parameters of f(x) are defined for a particular locality, community, or population, we can better engage communities with more relevant and relatable communications for risk messaging and subsequent increased compliance.^28^^,^^29^ So, Disease f(x) is a hierarchical mindset where the necessity of specific pathogen identity occurs on a continuum depending on which element of research, development, or response is being developed (Figure 1).

Although perhaps intuitive, the framework of Disease f(x) will broaden the mindset of stakeholders by explicitly representing that infectious disease risk is a sum of many parts. This has the added benefit of encouraging communication of uncertainties, as results are put into larger context, which in turn adds an element of transparency in our risk communication to the public, as absolutism—or even the appearance of absolutism—has confounded the current pandemic response.^23^ Adoption of this mindset across sectors, including biotechnology, disaster management, and epidemiology, will encourage development of more agile, efficient, and effective, but holistic preparedness and response programs to address the next major infectious threats.
